# Contrasting management regimes indicative of mesopredator release in temperate coastal fish assemblages

**DOI:** 10.1002/ece3.10745

**Published:** 2023-12-09

**Authors:** Ann‐Elin Wårøy Synnes, Esben Moland Olsen, Per Erik Jorde, Halvor Knutsen, Even Moland

**Affiliations:** ^1^ Centre for Coastal Research Department of Natural Sciences University of Agder Kristiansand Norway; ^2^ Institute of Marine Research, Flødevigen His Norway

**Keywords:** atlantic cod, functional status, marine protected areas, top predator, trophic cascade

## Abstract

The absence of functional top predators has been proposed as a mechanism acting to shape fish assemblages in temperate marine ecosystems, with cascading effects on lower trophic levels. We explore this scenario by comparing the trophic and functional status of fish assemblages in Norwegian marine national parks, open to fishing, to a nearby coastal seascape that harbors a system of marine protected areas (MPAs) including a no‐take zone. Demersal fish assemblages were sampled using fyke nets over three consecutive seasons. Atlantic cod (*Gadus morhua*) is potentially a dominant top predator in this ecosystem, and historically, this and other gadids have been targeted by the full range of former and present fisheries. In the present study, we find that average body size of the Atlantic cod was significantly larger in the zoned seascape compared to the unprotected areas (mean ± SD: 36.6 cm ± 14.38 vs. 23.4 ± 7.50; *p* < .001) and that the unprotected seascape was characterized by a higher abundance of mesopredator fish species. These observations are consistent with the hypothesis that the protection of top predators within MPAs aids to control the mesopredator populations and provides empirical support to the notion that the present state of many coastal fish assemblages is driven by mesopredator release linked to functional depletion of large top predators.

## Graphical abstract

In this study, we compared the trophic and functional status of coastal fish assemblages in three Skagerrak seascapes: two national parks, at the mouth of the Oslo fjord—open to fishing, and one adjacent seascape harboring a system of marine protected areas (MPAs), including a no‐take zone. Findings provide empirical support to the notion that the present state of many coastal fish assemblages is driven by mesopredator release linked to functional depletion of large top predators.

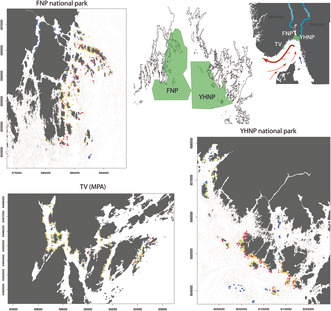

## 1 INTRODUCTION

Since the onset of the industrial fishing era, mean trophic levels of fisheries landings around the world have declined (Pauly et al., 1998). Apex consumers or top predators, defined as predators that occupy the higher trophic links in an ecosystem, may have a strong effect on the trophic dynamics and diversity of the system in which they occur (Baden et al., 2010; Moksnes et al., 2008). Reduction of large, piscivorous species can alter ecosystem productivity and result in cascading effects down the food web and thereby affect community structure as well as ecosystem functioning (Donadi et al., 2017; Steneck, 2012). In contrast, the historical view of the marine ecosystems was, to some extent, that the oceans were largely structured by bottom‐up control, meaning that the food web was mainly controlled by resource limitation (Cushing, 1975).

Even though primary producers and bottom‐up processes are influencing all marine food webs, recent studies have drawn attention to the importance of top predators and their role in the food web, potentially controlling populations of smaller predators (mesopredators), and grazers (Baden et al., 2012; Östman et al., 2016; Ritchie & Johnson, 2009). Overexploitation of larger top predators can lead to a dramatic increase of the lower trophic species that are present, where the magnitude of the cascade is dependent on several factors, such as the complexity of the food web (Eriksson et al., 2023). Among the most studied examples is the overexploitation of the sea otter (*Enhydra lutis*) in Alaska. The decline of the sea otter population lead to an increase of their sea urchin prey, which in turn left kelp forests destroyed due to overgrazing by the increased population of sea urchins. Trophic cascades caused by such top‐down control have been demonstrated in various ecosystems, as kelp forests (Estes et al., 2004), lakes (Persson et al., 2003), and streams (Bechara et al., 1992), as well as in oceanic systems (Baum & Worm, 2009; Frank et al., 2005; Myers & Worm, 2005; Shears & Babcock, 2002).

In recent decades, human activity has driven the functional extinction of many top predators, and several studies have indicated subsequent ecosystem changes that are complex and unpredictable (Ellingsen et al., 2015; Floeter et al., 2005; Frank et al., 2005). During the 1980s and 1990s, several Atlantic cod populations in the North Atlantic collapsed. In the same time period, Atlantic herring populations increased drastically (NEFSC, 1998). In the Baltic Sea, a collapse of the Atlantic cod populations was followed by an increase in abundance of the European sprat (Köster et al., 2003). It was hypothesized that predation on cod eggs and larvae from these lower level species might be a factor preventing the recovery of the cod populations (Köster et al., 2003). None of the cod populations in the Baltic Sea have recovered, even though fishing has been reduced (ICES, 2022). The less heavily harvested local cod population in the adjacent Öresund (The Sound) has retained broad size and age structure, also during periods of adverse environmental conditions (Lindegren et al., 2010; Sundelöf et al., 2013).

The Norwegian Skagerrak coastal system includes only a few higher trophic fish species, where Atlantic cod (*Gadus morhua*) is historically one of the most dominant top predators. During the last decades, however, there has been a substantial decline in the abundance of larger cod and other piscivorous fish in the North Sea and Skagerrak waters, as well as in Kattegat (Barceló et al., 2016; Rogers et al., 2017; Svedäng, 2003; Svedäng & Bardon, 2003). On the Swedish Skagerrak coast, current abundance of demersal fish >30 cm, including cod, in the inshore fish community is extremely low compared to historical records (Svedäng, 2003). The same decline of large cod (Perälä et al., 2020) and piscivorous fish has been observed along the Norwegian Skagerrak coast, especially in the eastern part of Skagerrak and the areas around outer Oslo fjord (IMR beach seine time series, unpublished). This has raised concern from both local and regional government, as well as among recreational and commercial fisheries located in this region (Jorde et al., 2018). The government is now raising the question if the stocks of local cod populations could be restored and brought back to the state they were in before the collapse observed in the early 2000s.

Using data collected over three survey years, our aim was to assess whether the absence of top predator species in the study system is the likely cause for an apparent mesopredator release. We do this by contrasting patterns in fish species composition, species richness, species abundance, and size distribution of top predators between two contrasting study areas along the Norwegian Skagerrak coast: (1) recently established marine national parks that are still open to fishing, and (2) a neighboring fjord in which there has been a decade‐long protection of fish within MPAs. As the Atlantic cod is considered the dominant predator in this region, it was our main focus, although other top predator species present in the system were also investigated. High abundance of mesopredatory fish in the exploited area motivated a further investigation into the relationship between the most abundant mesopredatory fish species, shorthorn sculpin, and Atlantic cod.

## 2 MATERIALS AND METHODS

### 2.1 Study areas

**FIGURE 1:**
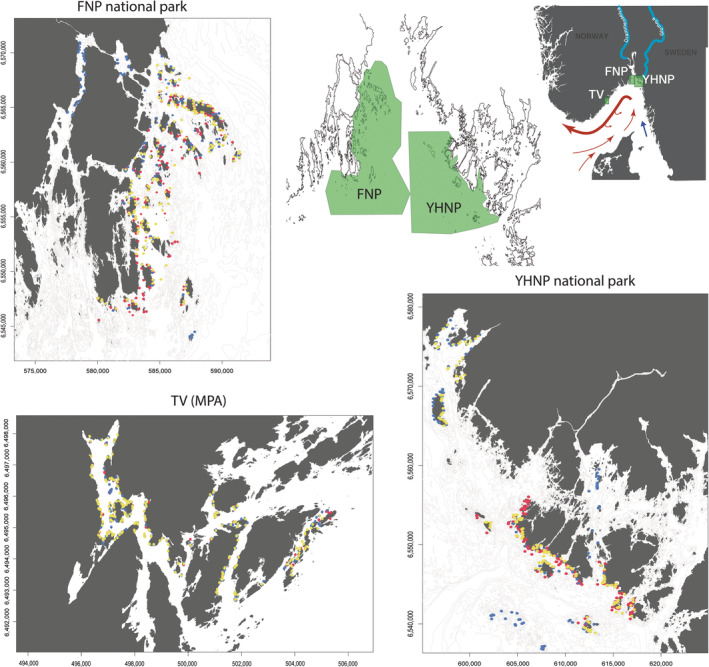
Map of Norwegian Skagerrak coast and sampling sites on east and west side of outer Oslo fjord, and the Tvedestrand zoned seascape located further south on the coast. Red dots represent sampling sites from 2017, blue 2018, and yellow 2019.

This study was conducted in the outer Oslo fjord during 2017, 2018, and 2019. In this area, two national parks were established to protect habitats and arrest development in the coastal zone: Ytre Hvaler National Park (YHNP, hereafter referred to as “East”) in 2009 and Færder National Park (FNP, hereafter referred to as “West”) in 2013, situated on the eastern and western side of the fjord mouth, respectively (Figure 1). Although the sampling areas hold a status as national parks, there were no special restrictions on fishing inside the parks at the time of study, with the exception of gear limitations (only hook‐and‐line gear allowed) in lobster reserves and prohibition of bottom‐towed gear on known cold water coral (*Lophelia* sp.) reefs in Ytre Hvaler national park. Until recently, a minimum size limit of 40 cm for cod caught within the 12 nm border was the only regulation for cod catch. In 2019, a recreational ban on cod fishing was implemented from Telemark County to the Swedish border, which also includes a seasonal ban on cod fishing at known coastal spawning sites. No other fish species of higher trophic levels are protected.

The area covered by our survey was approximately 200 km^2^ on the west side and 190 km^2^ on the east side of the fjord. The outer Oslo fjord seascape consists of archipelagos and several smaller fjords, bays, and estuaries. It is a relatively exposed area, which is influenced by several distinct water masses. The upper layers of the water column consist mainly of brackish water due to river discharge and inflow of brackish water from Kattegat and the Baltic Sea (<25.0 psu). Underneath this brackish water layer, there is a mixing of water masses from the North Sea and surface layer (25–35 psu), while high‐saline, nutrient‐rich, Atlantic water (>35 psu) flows up from the Norwegian Trench and is usually found at depths greater than 70–80 m.

The outer Oslofjord is considered as an eutrophicated area partly due to the increased supply of nutrients from Norway's two largest rivers, Drammenselva and Glomma, and also smaller river systems in the inner parts of the Oslofjord. Due to increased rainfall during the last decades, these rivers carry high amounts of soil particles, nitrogen, and phosphorous which are released into the sea (Walday et al., 2017). The outer Oslofjord area is also affected by long‐term fishing pressure, where both commercial and recreational fisheries have contributed strongly to the depletion of larger bodied piscivorous fish, including gadoids (Cardinale & Svedäng, 2004; Casini et al., 2005).

Tvedestrand municipality is situated 120 km southwest of the outer Oslofjord. In 2012, the Tvedestrand fjord and outer coastal areas were subject to a zoning process in which ≈15% of municipality waters were included in no‐take or partially protected areas (PPAs). For a detailed description of the zoning, see Moland et al. (2021). The Tvedestrand fjord proper is a small fjord including several sills and basins, extending approximately 8 km inland. It includes a great variation of habitats, such as eel grass beds, soft corals, mud flats, and kelp forests (Freitas et al., 2016). It also harbors inshore spawning aggregations and nursery areas for coastal cod (Ciannelli et al., 2010; Knutsen et al., 2007). In 2012, a 1.5‐km^2^ no‐take reserve was implemented in this fjord for protection of fish and lobsters against commercial and recreational fishing. This reserve effectively protects 40%–80% of the home ranges of at least two resident aquatic top predators: the anadromous brown trout and the Atlantic cod (Thorbjørnsen et al., 2019; Villegas‐Ríos et al., 2017). On each side of the no‐take zone is a partially protected zone, where only hook and line type gear are allowed. In the northeastern part of municipality waters, a 4.9‐km^2^ partially protected area extends from the outer islands to approximately 50 m depth. The Tvedestrand seascape covered by the fyke net survey (see below) measures approximately 17 km^2^ and has a topography that is representative of fjord‐to‐coast systems along the Norwegian Skagerrak coast (Figure 1). The inner fjord has a variable freshwater surface layer, below which the temperature and salinity increase with depth down to ~30 m (Ciannelli et al., 2010), whereas in the outer exposed areas, the freshwater layer is absent.

### 2.2 Sampling procedure

To assess the fish assemblage in Outer Oslofjord, sampling was performed on the eastern and western side of the fjord mouth, located approximately 20 km apart. Sampling was done using fyke nets with 55 cm openings and 25 mm mesh size. fyke net stations were chosen based on experiences gathered from fyke net surveys designed to sample gadoids in Tvedestrand fjord and beyond. With a considerably larger seascape covered in outer Oslofjord, we prioritized good geographical coverage of subareas and random fyke net placement within the constraints of suitable habitat depth and inclination. Fyke nets were deployed in gentle slopes or level habitat, with the cod‐end toward the deep, usually in depths <6 m. This experimental fishing was conducted in early May in 2017–2019. A total of 930 fyke nets were hauled during the three surveys, as well as 111 large collapsible baited fish traps (130 × 80 × 120 cm) for “control” sampling of deeper habitat (>10 m). Soak time was approximately 24 h for both fyke nets and traps. Catches were recorded directly on board, and all fish were counted and identified to species level and measured to nearest centimeter (fork length), before being released back into the sea. A tissue sample was collected from all *G. morhua* individuals for genetics analyses (to be reported elsewhere). After sampling, the fishing gear was relocated to a new position (chosen at random, but with criteria as explained above), before being hauled again the next day. In outer Oslo fjord, each site was sampled for 4 days except for 2018 when the eastern side was sampled for 3 days (Table S1). To be consistent from a taxonomic point of view, non‐fish organisms were excluded from the data analysis.

The protected seascape (Tvedestrand) was sampled using fyke nets in May for the years 2017, 2018, and 2019. A total of 606 fyke nets were deployed during the 3 years of sampling, following the same general procedure as in outer Oslofjord. Sampling was carried out for 6 days in 2017 and 2018, and 7 days in 2019. The fjord was sampled inside the no‐take‐ and partially protected zones, and also further out toward the exposed areas beyond the fjord mouth (Figure 1).

### 2.3 Statistical analysis

To compare the fish communities sampled in the outer Oslofjord and Tvedestrand seascapes, fish species' relative abundance (catch‐per‐unit‐effort; CPUE), representing densities of fish species (N/fyke nets/days) was calculated for both juvenile and adult life stages for the most abundant families. For each sampling year, the Shannon diversity index, Simpson index, and species evenness were calculated for all sampling sites to assess differences between sites and years. In addition, sampling sites were clustered into 11 and 12 different clusters following a north–south gradient, where cluster No. 1 was situated in the northern part of the national parks and No. 12 in the southernmost part. This clustering was done to explore patterns in occurrence of species on the eastern and western side of outer Oslo fjord, as well as potential ecosystem “hot spots.” Clustering was done under the assumption that islands and land areas close to each other would have somewhat similar fish assemblages (Table S3). Shannon and Simpson diversity indexes and evenness were also calculated for the same 11 and 12 clusters (Table S3). The degree of similarity in frequencies of all species found in the two national parks and adjacent areas was calculated using a heatmap of the Jaccard similarity index using the package pheatmap (Kolde, 2022), with dendrograms showing similarity between species abundance, sites, and years (Figure S1).

We applied linear models (McCullagh, 2018) to compare species diversity indexes and evenness between the three sampling regions (Tvedestrand, FNP, and YHNP) and years. Plotting the raw data indicated similar variance among regions in each sampling year, with seemingly shared year‐to‐year differences. We thus chose to run models with an interaction effect between region and sampling year, with year modeled as a factor. Residual plots indicated that models fitted the data adequately. We tested for an effect of region and sampling year on species diversity (Shannon and Simson indexes) using the following model structure:

(1) Diversity=Region×Year

The same model structure was used to test for an effect of region and sampling year on evenness.

Generalized linear models (McCullagh, 2018) were used to investigate effects of contrasting management regimes on cod abundance and average body size. Preliminary analyses showed that a large proportion of the fyke net hauls did not contain any cod. Therefore, cod catch (*CC*) was analyzed as a binary process (i.e., the probability of catching at least one cod per fyke net). Sampling year was added as a factor to control for temporal variation in catches:

(2) CC=Region×Year

Next, we used the same model structure to test for effects of contrasting management regimes on the presence of above legal‐size cod (>40 cm).

A model without explanatory variables (null model) was fitted to test the hypothesis that none of the variables influenced the abundance or size of the top predators. Both variables “region” and “year” was also tested separately. A model selection based on Akaike's information criterion (AIC) was used to determine the most parsimonious model, and the model with the lower AIC was selected as the best one.

Differences in size distribution for top predator species between outer Oslofjord and the MPA were tested with a Welsh two‐sided *t*‐test. All data analyses were conducted using the open‐source language R 3.6.1 (R Core Team, 2019), using the package vegan for calculation of diversity indexes (Oksanen et al., 2019).

Each fish species was assigned a trophic level using information from FishBase (www.fishbase.org) and grouped into categories as low‐, mid‐, or high‐level carnivore. Low‐level carnivores were identified as species with a trophic level ranging from 3 to 3.5, and mid‐level carnivores were identified as species with a trophic level ranging from 3.5 to 3.9. Predators grouped into high‐level carnivores were identified as species with a trophic level ≥ 4.0 (as done in Essington et al., 2006), hence including the gadoids cod, saithe, whiting and pollack, as well as the species from the Scopthalmidae family, garfish and great weever. To test if there was a difference in proportions of the trophic level species between the exploited area in outer Oslo fjord and the protected area of Tvedestrand, we used a two proportion Z‐test with Yates' continuity correction for small expected values (prop.test in R).

Life stage categories of adult and juvenile were based on the species‐specific length at maturity according to FishBase (Froese & Pauly, 2016; Staveley et al., 2017). For species where maturity data were unobtainable, an alternative method commonly used to determine life stage was applied, where individuals that were ≤1/3 of their maximum length (according to FishBase) were recorded as juveniles (Dorenbosch et al., 2006; Nagelkerken & Van der Velde, 2002; Staveley et al., 2017).

## 3 RESULTS

### 3.1 Fish assemblage in outer Oslofjord

**FIGURE 2:**
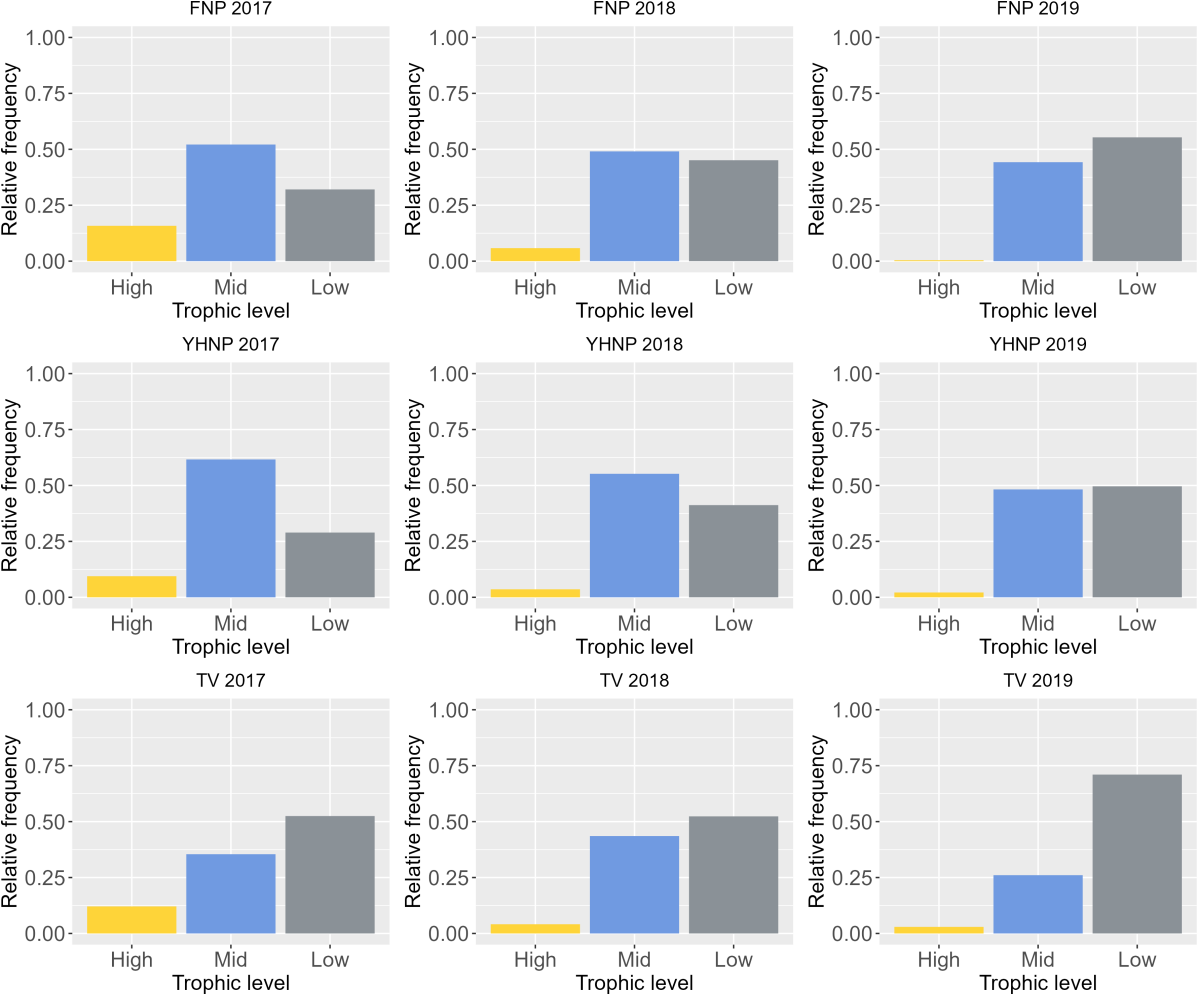
Relative frequency of trophic levels for all fish species caught in the outer Oslo fjord and in the Tvedestrand zoned seascape during 3 years of sampling. Abundance of high‐level carnivores is displayed as yellow bar, mid‐level carnivores is displayed as blue bar, and low‐level carnivores are displayed as gray bar.

**FIGURE 3:**
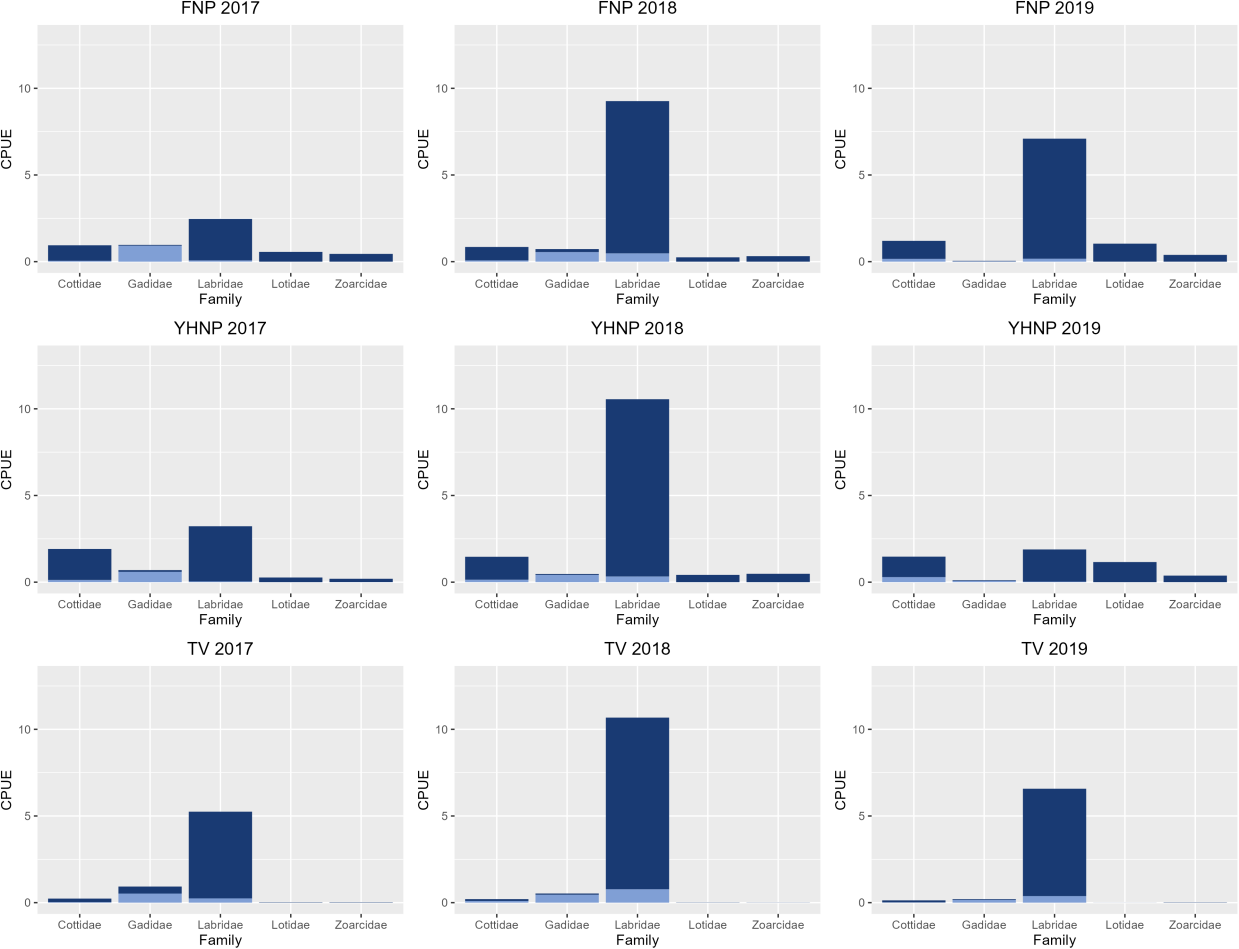
CPUE (N/total fyke nets/days) split into life stages for the most common taxonomic groups present at both national parks (FNP, YHNP) and Tvedestrand (TV) for all years of sampling. Dark blue bars represent CPUE of adults, while light blue bars represent CPUE of juveniles.

**FIGURE 4:**
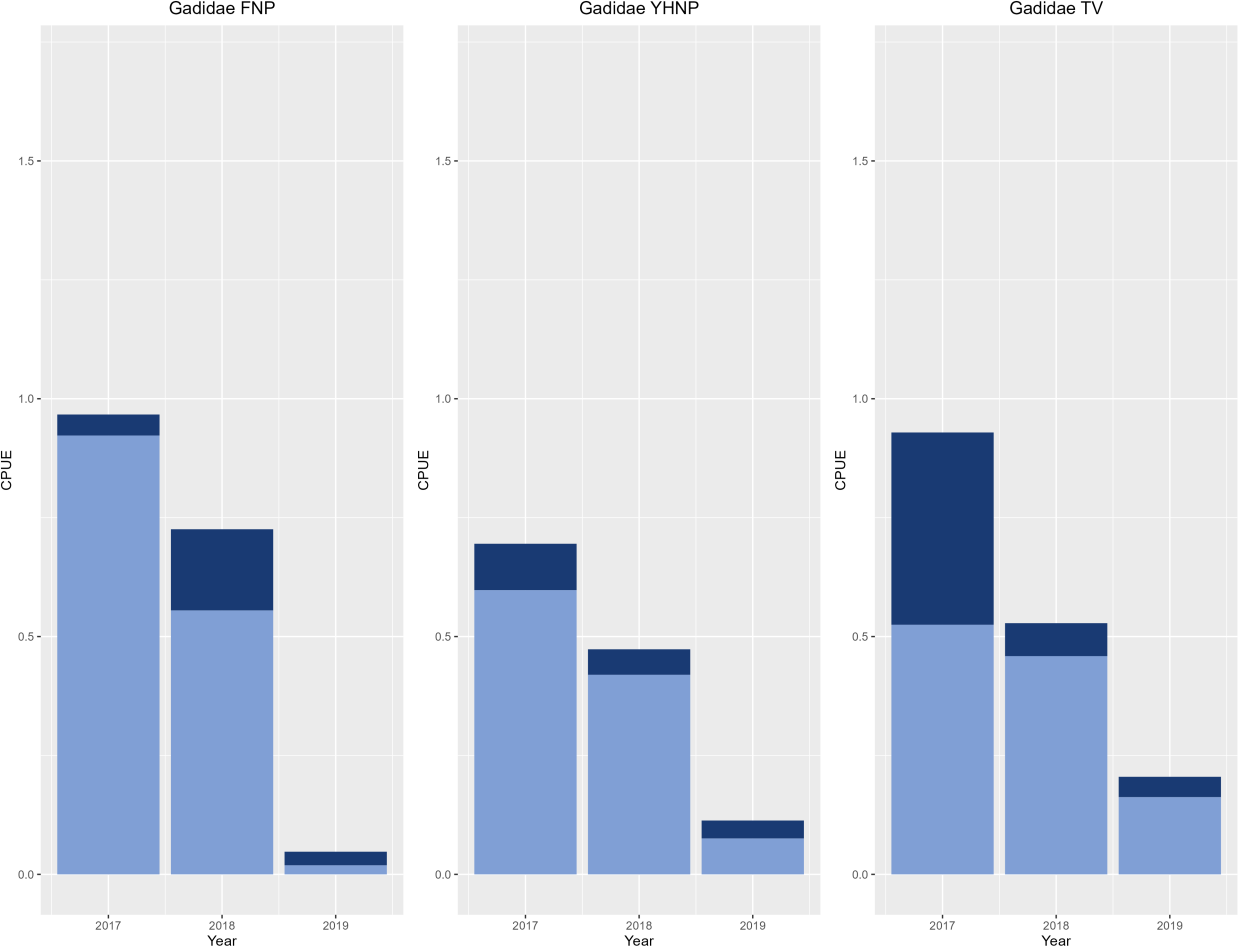
CPUE (N/total fyke nets/days) for the Gadidae family, representing the group holding the most important top‐predator species for the study areas, present at both national parks (FNP, YHNP) and Tvedestrand (TV) for all years. Dark blue bars represent CPUE of adult individuals while light blue bars represent CPUE of juveniles.

A total of 7959 individual fish comprising 34 species from 19 families were recorded from 930 fyke net hauls at the east and west side of outer Oslo fjord. Of these, most species were classified as mesopredators (Table S2, Table 1, Figure 2), where the Labridae and Cottidae families had the highest abundances throughout all years of sampling (Figure 3). Atlantic cod accounted for 11% of the fish community (by numbers) in 2017, 3.2% in 2018, and only 0.8% in 2019 (Figure 4). Notably, most of the gadids were small juveniles (Figure 4). In contrast, the dominating mesopredators were mostly classified as adult individuals (Figure 3).

**TABLE 1:** Sample overview from all fyke net hauls performed during 3 years of sampling, displaying total sample size (all fish caught), mesopredator (all individuals assigned to low‐ and mid‐level carnivores), top predator, and cod abundance from all years of sampling.

Region	Year	Total sample size	Mesopredator		Top‐predator	Atlantic cod
			Low	Mid		
FNP	2017	1076	345	561	170	156
	2018	2184	946	1125	113	75
	2019	1216	673	538	5	3
YHNP	2017	1230	356	758	116	97
	2018	1598	652	893	53	42
	2019	649	322	314	14	12
Total		7953	3294	4189	471	385
Tvedestrand	2017	1067	560	378	129	66
	2018	3159	1653	1376	130	25
	2019	1808	1284	471	53	14
Total		6034	3497	2225	312	105

The additional sampling of deeper areas in outer Oslo fjord using baited fish traps yielded a total of 304 individual fish comprising 10 species from six different families. The deeper areas also had a higher frequency of mesopredators, where the Pleuronectidae family had the highest abundance throughout all years, with common dab (*Limanda limanda*) as the most abundant species.

### 3.2 Fish assemblage in the zoned seascape—Tvedestrand

In the Tvedestrand fjord and adjacent areas, a total of 6035 individual fish comprising 34 species from 16 families were registered from 606 fyke net hauls. Most species were classified as mesopredators, where Labridae was the most abundant family in for all years, with corkwing and goldsinny wrasse as the most dominant species (Figure 3).

### 3.3 Species richness, evenness, Shannon and Simpson index

Fish communities showed similar patterns between east and west side of outer Oslo fjord for the years 2017 and 2018; however, the eastern side had generally lower abundances of fish in the 2019 hauls (Table 1, Figure 3). Average species richness was highest on the western side of the Oslo fjord and lowest on the eastern side (Table S4). Tvedestrand had equal richness for all years of sampling (Table S4). Results from samples clustered into 11 or 12 sites based on a north–south gradient showed little difference in Shannon or Simpson indexes or Evenness and appeared similar (Table S3). The linear model (equation 1) test results showed that neither the Shannon nor Simpson diversity indexes were different between the three sampling regions Tvedestrand, eastern or western outer Oslo fjord (Tables S5 and S6). However, all regions shared somewhat lowered Shannon and Simpson (*p* < .05) indexes in 2019. Species Evenness ranged from 0.56 to 0.81 among years and areas (Table S4), where the eastern side of outer Oslofjord had the overall highest Evenness and the western side of outer Oslofjord had lower Evenness overall (Table S4). The linear model supported an effect of region on species diversity only for 2019, with significantly lower Evenness for the western outer Oslo fjord (Table S7).

### 3.4 Comparison of outer Oslofjord and Tvedestrand

**FIGURE 5:**
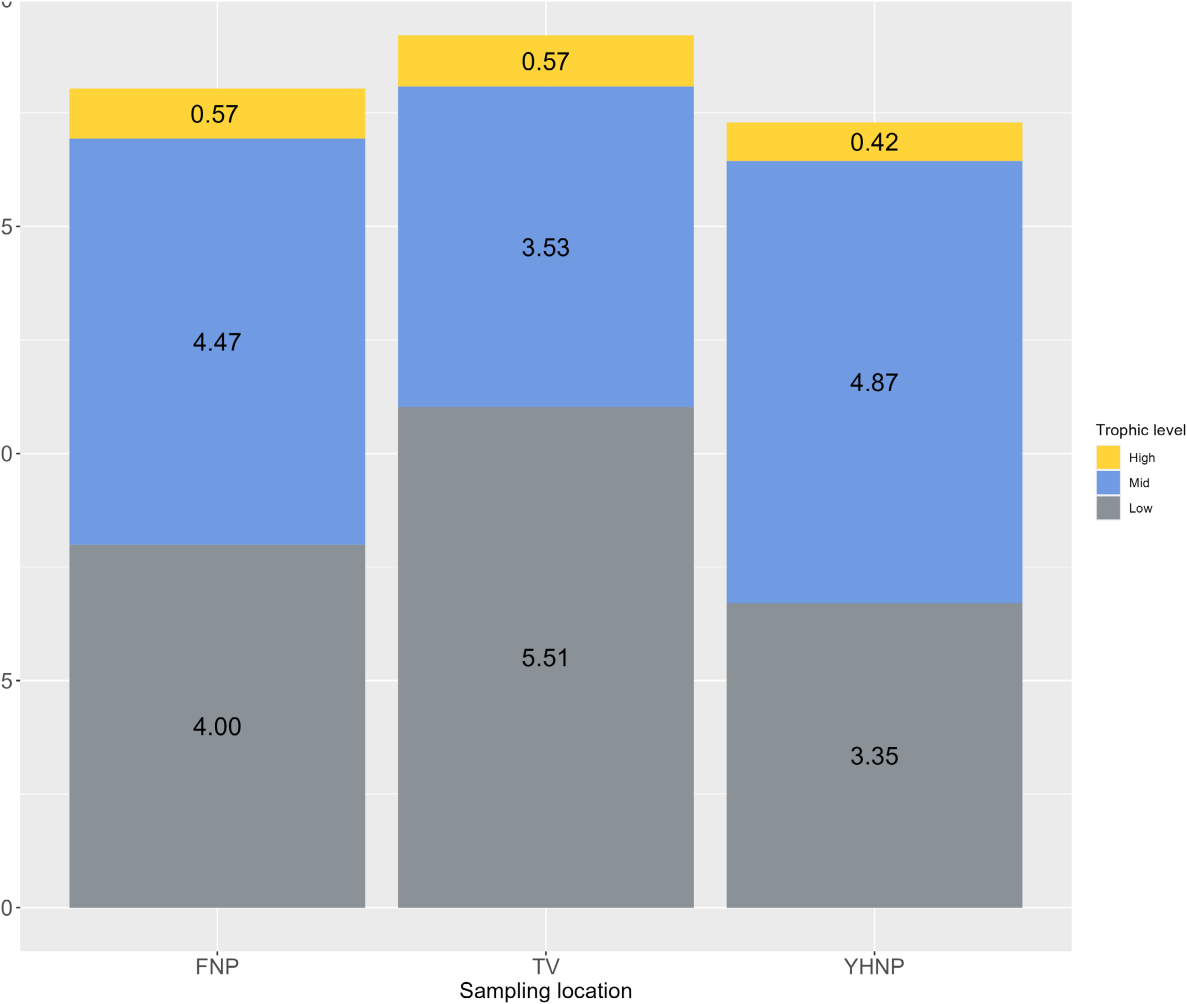
Mean CPUE (N/total fyke nets/days) for all species caught from the three sampling sites and all years grouped into low‐ (gray), mid‐ (blue), and high‐level (yellow) carnivores based on diets (www.fishbase.org).

Density of fish species assigned to trophic level (low‐, mid‐, and high‐level carnivores) varied considerably among years for the sample sites in outer Oslo fjord, and less so in Tvedestrand (Figure 2). Low‐level carnivores dominated the catches in most years, especially in the Tvedestrand seascape, while mid‐level carnivores showed a higher abundance in the fished area in Oslo fjord than in the protected area in Tvedestrand (Figure 2, Figure 5, Table S2). High‐level carnivore species had the highest abundance in 2017 for all sampling locations (Figure 2, Table S2). A two proportion *Z*‐test showed that there was a significantly greater proportion of mid‐level carnivores (*p* < .001), and less low‐level carnivores in outer Oslo fjord compared to Tvedestrand (*p* < .001). However, there was no significant difference in proportions of top predators between the outer Oslofjord and Tvedestrand (χ^2^ = 3.52, df = 1, *p* = .06).

**FIGURE 6:**
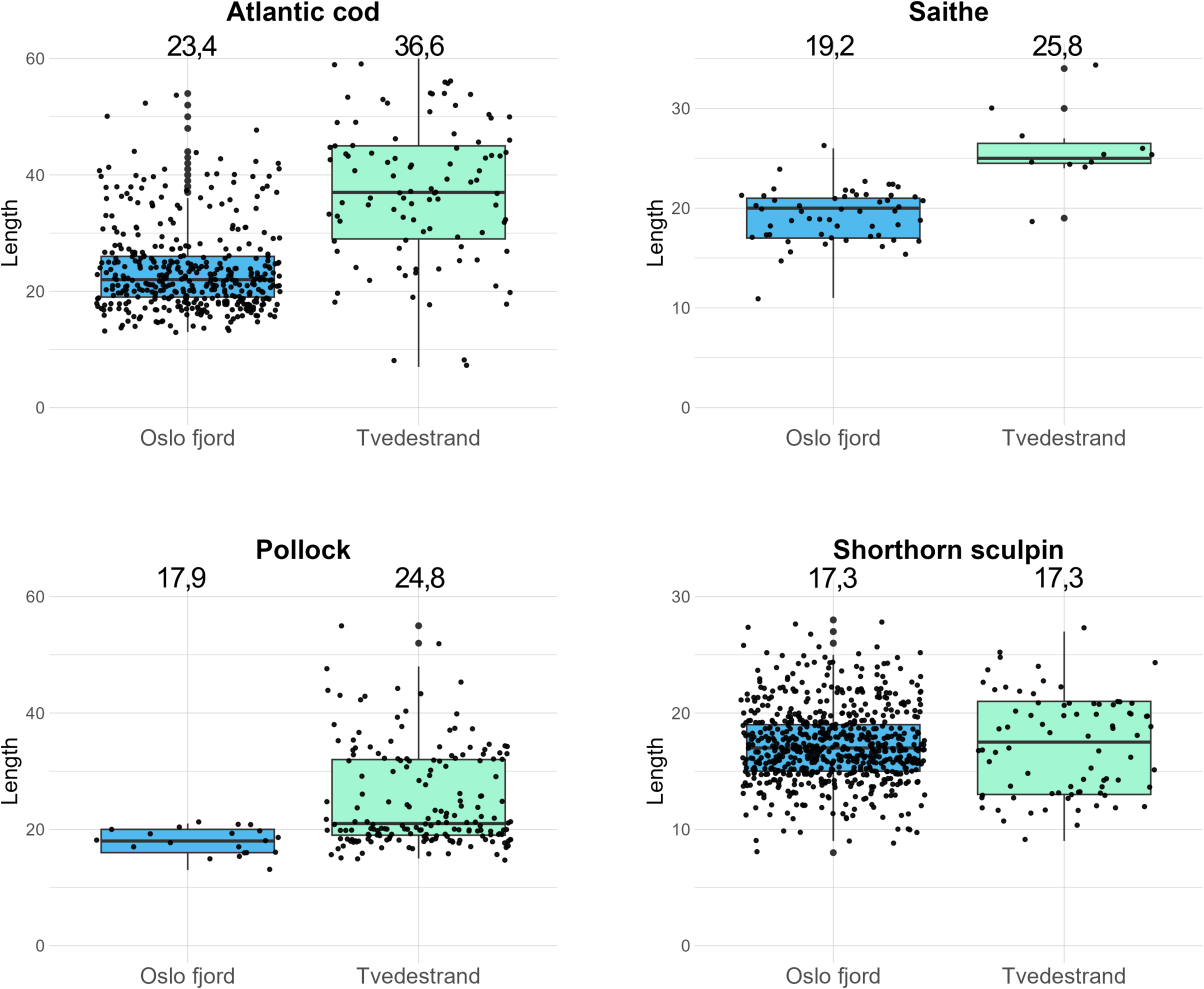
Length distribution for top‐predator species Atlantic cod (*Gadus morhua*), saithe (*Pollachius virens*), pollack (*Pollachius pollachius*), and shorthorn sculpin (*Myoxocephalus Scorpius*) from samplings performed in Tvedestrand and outer Oslo fjord (samplings from both national parks pooled together) for all catches collected in 2017, 2018, and 2019. Box displays a confidence interval around the median, while average length is noted above the boxplots.

For key predatory fish species, body size was on average 62%, 30%, and 34% greater in the Tvedestrand seascape compared to the outer Oslo fjord national parks for Atlantic cod, pollack, and saithe, respectively (Figure 6). Welch two‐sided *t*‐tests confirmed significant differences in mean top predator species body lengths between Tvedestrand and outer Oslo fjord (Atlantic cod: *t* = −9.06, *df* = 118.94, *p* < .001; Pollack: *t* = −8.58, *df* = 94.13, *p* < .001; Saithe: *t* = −5.51, *df* = 11.99, *p* < .001). For Atlantic cod, the 90th percentile length was 35 cm in outer Oslo fjord compared to 54 cm in Tvedestrand.

Atlantic cod accounted for 80% of the catch of top predators in outer Oslo fjord and 33% in Tvedestrand (Table 1). The best model for predicting the presence of cod (equation 2) supported a regional effect that also varied among years (i.e., a region × year interaction term, table in Appendix A). Overall, fyke net hauls in the Tvedestrand seascape had a lower probability of cod catch compared to the national parks (Table S8). There was a significant decrease in cod catch for 2018 and 2019 (*p* < .001) (Table S8). Also, the western side of outer Oslo fjord had a higher abundance of cod in 2018, and lower abundances in 2019, compared to the 2017 sampling (*p* < .01) (Table S8). The eastern side of outer Oslo fjord had a higher abundance of cod in 2018 compared to 2017 (*p* < .05); however, no difference was found for 2019 (Table S8). For cod above the legal‐size limit (>40 cm), both eastern and western side of the outer Oslo fjord had a significantly lower abundance of cod in 2017 compared to Tvedestrand (Table S9). The western side of outer Oslo fjord had a significantly higher abundance of cod above legal‐size limit compared to Tvedestrand in 2018 and in 2019. Compared to Tvedestrand, the eastern side of the fjord had significantly higher abundance of cod above legal‐size limit in 2018 but not 2019 (cf. Table S9).

## 4 DISCUSSION

By comparing disparately managed coastal regions, this study provides empirical support for a mesopredator release linked to depletion of large top predators in temperate fish communities. Specifically, top predators such as the Atlantic cod had consistently larger body size in the zoned/ partially protected region, even though abundance was variable among years. The unprotected regions saw consistently higher abundances of top predator prey, that is, mesopredator fishes such as labrids. We discuss our findings against the potential for restoration of top predator abundance and size structure, and recovery of the former species assemblage.

Diversity indices indicated highly similar species assemblages in the two study areas. While we are aware of the work by Jost (2006) calling for the use of effective numbers rather than diversity indices—we chose to apply the classical framework in the present study.

There were more species assigned as high‐level predators in the Tvedestrand seascape samples compared to outer Oslo fjord. The most abundant ones were pollack and Atlantic cod, while several of the other top predator species only occurred once or twice in the fyke net hauls. This overall result suggests that there is a greater diversity of top predators in the zoned area. We note, however, that our sampling approach may be biased when it comes to larger species. Sedentary species may be poorly sampled as the fish needs to swim into the net and are not actively targeted. Also, larger fish may utilize a greater variety of habitats and only frequent the nearshore habitats on a seasonal or diurnal basis (Freitas et al., 2021).

The lower abundance of mid‐level carnivore fish in the protected fjord indicates that the higher trophic level species in this area still play a functional role in the ecosystem by limiting their prey. Earlier research has argued that removal of top predator species from complex marine food webs with many interacting species may weaken the top‐down effects, and trophic cascades arise only in simple food webs lacking functional redundancy (Donadi et al., 2017; Shurin et al., 2002). Our results show that the fish assemblage in outer Oslo fjord contain a higher abundance of mid‐level carnivores compared to the Tvedestrand seascape. Also, we show that the abundance of mid‐ and low‐level carnivores was more stable in the protected Tvedestrand seascape, whereas it showed more variability among years in the fished regions in the outer Oslo fjord. These results could indicate that outer Oslofjord is suffering from a trophic level dysfunction, where the large top predators (with Atlantic cod being the dominant species) have been largely extirpated, and the mesopredatory fish species have subsequently taken over their trophic niche (Bourque et al., 2008; Floeter et al., 2005).

In concordance with our results, there is a general expectation of an increase in abundance of larger predatory species within MPAs or lightly fished areas, compared to exploited areas (Claudet et al., 2006; Friedlander & DeMartini, 2002; Watson et al., 2007), as well as an increase of lower trophic‐level species in ecosystems experiencing substantial declines of top predator species (Eriksson et al., 2011; Friedlander & DeMartini, 2002). When larger predators disappear, the ecosystem typically responds with an increase in densities of smaller predatory fish species, and with it follows marked changes in ecosystem structure and function (Jackson et al., 2001).

Our study found considerable variation in Atlantic cod presence during 3 years of sampling in both outer Oslo fjord and Tvedestrand seascapes. This is not unexpected. It is well known from previous analyses of time‐series data that there is high natural variability in Atlantic cod recruitment and presence, also in southern Norway (Johannessen et al., 2012; Smith & Page, 1996; Stenseth et al., 2006). This variability could be linked to external drivers such as temperature and local fishing pressure (Fernández‐Chacón et al., 2015; Rogers et al., 2017) as well as density‐dependent factors such as cannibalism and competition (Bjørnstad et al., 1999).

We acknowledge that the outer Oslofjord and the Tvedestrand fjord are different seascapes, not to be considered as randomized study units. The outer Oslofjord is a more exposed area compared to the partially sheltered Tvedestrand seascape, as well as situated at a somewhat higher latitude. In addition, the area sampled in Tvedestrand was considerably smaller (17 km^2^) than the two national parks sampled in outer Oslo fjord (FNP: 300 km^2^, YHNP: 190 km^2^). However, these areas are fundamentally similar in topography and geology. They are exposed to the same coastal current flowing east to west in Skagerrak. Thus, although some of the variation in species abundance and occurrence in our data could be due to different seascape properties, it seems likely that significant differences in top‐predator size are mainly due to high fishing pressure, as have been found in the eastern Skagerrak (Baden et al., 2010; Christie et al., 2020; Sköld et al., 2022; Svedäng, 2003).

Notably, the shorthorn sculpin (*M. scorpius*) and long‐spined bullhead (*T. bubalis*) where highly abundant in both sampling areas in outer Oslofjord. These cottid species are known to be piscivore hunters, and especially the shorthorn sculpin is known for being capable of eating fish almost as big as its own body size. Results from the nearby Swedish west coast by Wennhage and Pihl (2002) indicate that, depending on habitat, there might be intraguild competition for the resources shared between the Atlantic cod, shorthorn sculpin, and longspined bullhead (see also Dunlop et al., 2022). Although no diet analysis was done in the present study for the sculpins and Atlantic cod in outer Oslofjord, we did find a positive association between the species in outer Oslo fjord that was not seen for the Tvedestrand seascape. The high abundance of shorthorn sculpin we observed in outer Oslo fjord could be a factor negatively affecting the cod populations recruitment success by predating on eggs and larvae, as well as newly settled young of the year (0 group) cod. Predation mortality from shorthorn sculpin has previously been reported to be higher than from Atlantic cod and saithe (Pedersen et al., 2020). The high abundance of this species might thus represent yet another impediment for the Atlantic cod to redeem its place as a top predator in this ecosystem.

Fish stock collapses can result in large changes to marine ecosystems, as trophic cascades and eventually regime shifts that span over multiple tropic levels and can alter the energy flow in the system (Donadi et al., 2017; Pershing et al., 2015). Concurrent with the decline of Atlantic cod and other piscivorous fish >30 cm on the Swedish Skagerrak west coast (Svedäng, 2003), the abundance of mesopredatory fish such as gobids and labrids has increased in coastal Skagerrak (Barceló et al., 2016; Bergström et al., 2016; Eriksson et al., 2011). Since the early 1990s, several cod stocks in the northwest Atlantic have experienced a collapse and has failed to respond to complete cessation of fishing (Frank et al., 2005). The recent implementation of restrictions on cod fishing along the Norwegian Skagerrak coast (including outer Oslofjord) could potentially have a positive effect on restoration of local cod populations. However, if the abundance of mesopredatory fish continues to increase, this might too delay cod recovery in this area.

Implementing larger MPAs could be a possible solution to improve ecosystem functions in Skagerrak, as an increase of larger top predator species could aid to suppress lower trophic groups once the predator populations are recovering. To date, such management actions are rare in the region. One exception is the 426 km^2^ no‐take zone in Kattegat, closed to fishing since 2009. Effects were recently evaluated by Sköld et al. (2022) and showed recovery of biomass and abundance of the local fish assemblage. Cod showed signs of recovery, but the effect was not significant which was explained by the intense fishing pressure exerted on the local cod population when moving beyond the limits of the no‐take zone, that is, the MPA is too small to be effective for this species. In the adjacent Öresund (The Sound), a de facto ban on bottom trawling in effect since 1932 has allowed cod to prosper, and the population maintained broad size and age structure also during periods of adverse environmental conditions (Lindegren et al., 2010; Sundelöf et al., 2013). Implementing MPAs in areas that are showing signs of ecosystem dysfunctions have recently shown promising results (Kraufvelin et al., 2022; Soler et al., 2015), especially for top predator abundance (Colléter et al., 2012; García‐Rubies et al., 2013). As greater diversity in species result in more complete food webs (Rooney et al., 2006; Worm & Duffy, 2003), MPAs offer better prey choices and availability which leads to increased abundance and better diet composition of species (Dell et al., 2015). Greater phenotypic diversity (see Fernández‐Chacón et al., 2020) of protected species may also confer ecosystem benefits, reinforcing the effect of functional roles changing throughout ontogeny and lifetime of long‐lived, large‐bodied species often absent from heavily harvested seascapes.

In conclusion, the findings reported herein suggest an increased proportion of larger individuals of top predator species as a putative effect of reduced fishing pressure and lower abundance of mesopredatory species as a result of higher predation inside and around the MPAs/PPAs in the Tvedestrand seascape. This study provides empirical support to the notion that the present state of many coastal fish assemblages is driven by mesopredator release linked to functional depletion of large top predators.

## AUTHOR CONTRIBUTIONS

**Ann‐Elin Wårøy Synnes:** Conceptualization (equal); data curation (lead); formal analysis (equal); funding acquisition (supporting); investigation (equal); methodology (equal); project administration (supporting); writing – original draft (lead); writing – review and editing (lead). **Esben Moland Olsen:** Conceptualization (equal); formal analysis (supporting); investigation (equal); methodology (equal); resources (supporting); validation (equal); writing – original draft (supporting); writing – review and editing (equal). **Per Erik Jorde:** Conceptualization (supporting); methodology (supporting); supervision (supporting); validation (equal); visualization (equal); writing – original draft (supporting); writing – review and editing (equal). **Halvor Knutsen:** Conceptualization (equal); project administration (supporting); supervision (supporting); validation (equal); writing – original draft (supporting); writing – review and editing (equal). **Even Moland:** Conceptualization (lead); data curation (equal); formal analysis (equal); funding acquisition (lead); investigation (lead); methodology (equal); project administration (lead); supervision (lead); writing – original draft (equal); writing – review and editing (equal).

## FUNDING INFORMATION

The research was supported by grant no. 294926 (CODSIZE) awarded by the Research Council of Norway (RCN), and by the Interreg/EU in the MarGen II project. Scientific cruises to outer Oslofjord were funded through the IMR coastal ecosystem research program and supported by RCN through an RFF Oslofjordfondet grant (No. 272090) to the “Krafttak for Kysttorsken” project.

## CONFLICT OF INTEREST STATEMENT

The authors declare that they have no known competing financial interests or personal relationships that could have appeared to influence the work reported in this paper.

### OPEN RESEARCH BADGES

This article has earned an Open Data badge for making publicly available the digitally‐shareable data necessary to reproduce the reported results. The data is available at [[https://doi.org/10.5061/dryad.vmcvdnd06]].

## Supporting information


Data S1.

## Data Availability

The data that support the findings of this study are openly available in DRYAD archives at https://doi.org/10.5061/dryad.vmcvdnd06. For review: https://datadryad.org/stash/share/4FJLdgQGre1hk1eZFIn0l6B8fT7OsAHOL69QCteblbo.

## APPENDIX A

### Explanation of code used for GLM in the paper: Contrasting management regimes indicative of mesopredator release in temperate coastal fish assemblages

The glm model used in this paper was used to compare species diversity indexes and evenness, as well as the effects of contrasting management regimes on cod abundance and average body size.

Since a large proportion of the fyke nets did not contain any cod, the cod catch was analyzed as a binary process (i.e., the probability of catching at least one cod per fyke net). Sampling year was added as a factor to control for temporal variation in catches:

$$\begin{array}{l} \mathrm{glm}(\text{Presence}\sim {\text{Region}}^{*}\text{factor}\left(\text{Year}\right),\\ \text{data}=\text{catch}\left[\text{catch\$Species}=={}^{"}{\text{Gadus morhua}}^{"}\right],\\ \text{family}={}^{"}{\text{binomial}}^{"}) \end{array}$$

The code was run with R studio 3.6.1.

The package “vegan” was used for calculating diversity indexes.

A model selection based on Akaike's information criterion (AIC) was used to determine the most parsimonious model (in bold), and the model with the lower AIC was selected as the best one:

**Table:**

Top‐predator species	Models	AIC
Cod	**GLM = Presence ~ Region * factor(Year)**	**1335.3**
	GLM = Presence ~ Region + factor(Year)	1350.6
	GLM = Presence ~ Region	1592.6
	GLM = Presence ~ factor(Year)	1373.5
0‐model	GLM = Presence ~ 1	1730.1
Cod >40 cm	**GLM = Presence ~ Region * factor(Year)**	**754.9**
	GLM = Presence ~ Region + factor(Year)	770.1
	GLM = Presence ~ Region	915.9
	GLM = Presence ~ factor(Year)	947
0‐model	GLM = Presence ~ + 1	1034.4
