# Supplementary material for: Contrasting management regimes indicative of mesopredator release in temperate coastal fish assemblages
Source: Ecol Evol. 2023 Dec 9;13(12):e10745. doi: 10.1002/ece3.10745 (PMC10710310; doi:10.1002/ece3.10745)
Supplement: Supplementary file 1 — Data S1. [file ECE3-13-e10745-s001.docx]

**Electronic Supplement**


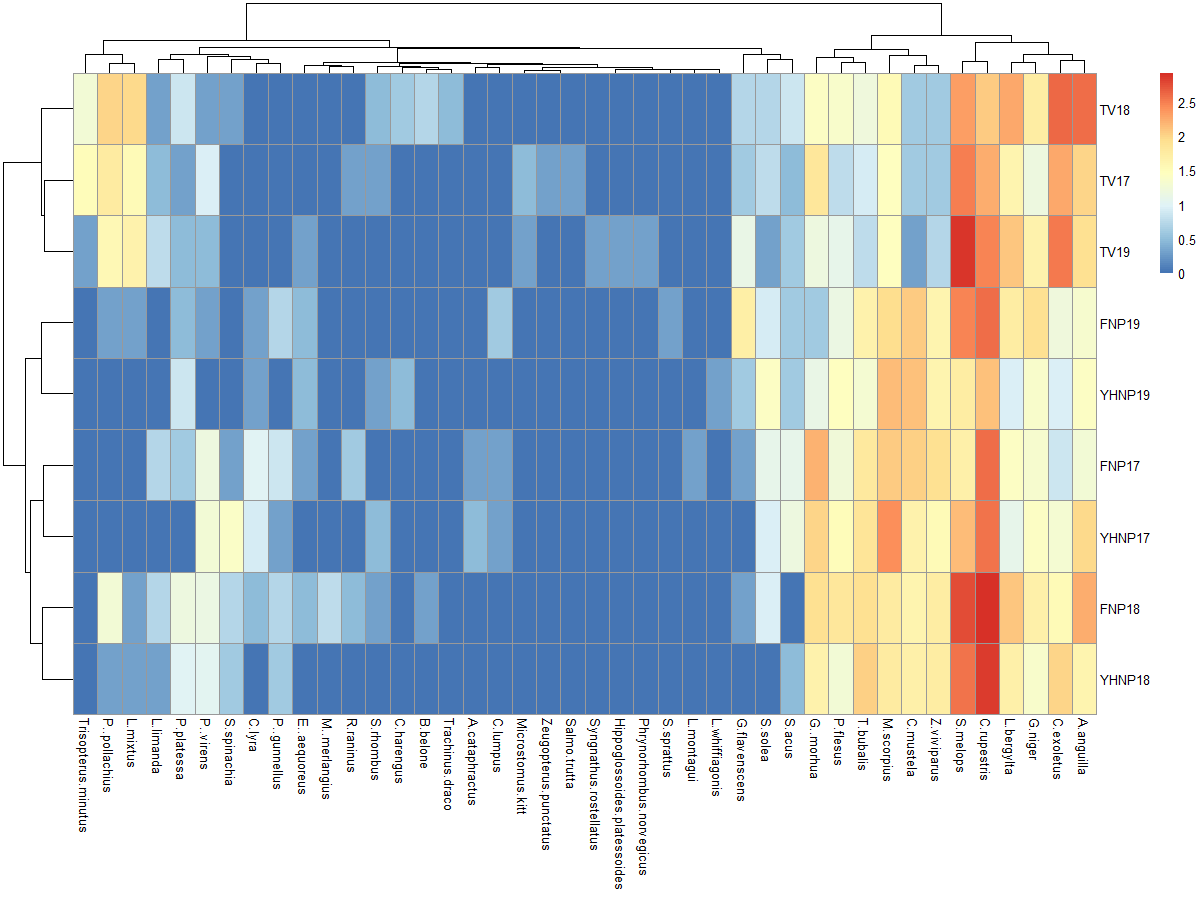


**Figure S1** Heatmap of frequency of species caught in fyke nets (log10 transformed) located in outer Oslo fjord (FNP, YHNP) and the Tvedestrand zoned seascape (TV) for all years. Color indicate strength of the relationship between sampling area/ year and abundance of the different species. Top dendrogram shows which species appeared most similar, side dendrogram shows which years appeared most similar.

**Table S1**. Summary of sampling years, total catch and number of fyke nets and traps used in the two national parks and in Tvedestrand

| **Sample site** | **Year** | **Fyke nets** | **Trap** | **Sampling days** | **Total catch (all species)** |
| --- | --- | --- | --- | --- | --- |
| **Færder national park** | 2017 | 181 | 21 | 4 | 1076 |
| **(FNP)** | 2018 | 159 | 20 | 4 | 2184 |
|  | 2019 | 156 | 20 | 4 | 1216 |
| **Ytre Hvaler National park** | 2017 | 166 | 19 | 4 | 1230 |
| **(YHNP)** | 2018 | 112 | 14 | 3 | 1598 |
|  | 2019 | 156 | 17 | 4 | 649 |
| **Tvedestrand** | 2017 | 141 | 0 | 6 | 1067 |
| **(TV)** | 2018 | 231 | 0 | 6 | 3159 |
|  | 2019 | 234 | 0 | 7 | 1808 |
| **Total** |  | **1536** | **111** | **42** | **13987** |

**Table S2.** List of species caught in fyke nets in outer Oslo fjord and the Tvedestrand zoned seascape assigned to trophic level and -category. Low = low-lever carnivore, Mid = mid-level carnivore, High = high-level carnivore. Information on species specific trophic levels and standard errors (SE) derived from fishbase.org.

| **Family** | **Species** | **Common name** | **Trophic level** | **SE** | **Trophic category** |
| --- | --- | --- | --- | --- | --- |
|  |  |  |  |  |  |
| Gadidae | *Gadus morhua* | Atlantic cod | 4.1 | ±0.2 | High |
|  | *Merlangius merlangius* | Whiting | 4.4 | ±0.2 | High |
|  | *Pollachius virens* | Saithe | 4.3 | ±0.3 | High |
|  | *Pollachius pollachius* | Pollack | 4.3 | ±0.4 | High |
|  | *Raniceps raninus* | Tadpole fish | 3.8 | ±0.56 | Mid |
|  | *Trisopterus minutus* | Poor cod | 3.7 | ±0.2 | Mid |
| Labridae | *Centrolabrus exoletus* | Rock cook | 3.5 | ±0.50 | Low |
|  | *Labrus bergylta* | Ballan wrasse | 3.2 | ±0.0 | Low |
|  | *Symphodus melops* | Corckwing wrasse | 3.4 | ±0.1 | Low |
|  | *Ctenolabrus rupestris* | Godsinny-wrasse | 3.6 | ±0.2 | Mid |
|  | *Labrus mixtus* | Cuckoo wrasse | 3.9 | ±0.62 | Mid |
| Pleuronectidae | *Limanda limanda* | Common dab | 3.4 | ±0.64 | Low |
|  | *Platichthys flesus* | European flounder | 3.3 | ±0.2 | Low |
|  | *Hippoglossoides platessoides* | American plaice | 4.1 | ±0.0 | High |
|  | *Pleuronectes platessa* | European plaice | 3.2 | ±0.50 | Low |
|  | *Microstomus kitt* | Lemon sole | 3.2 | ±0.33 | Low |
| Scopthalmidae | *Scophthalmus rhombus* | Brill | 4.4 | ±0.1 | High |
|  | *Lepidorhombus whiffiagonis* | Megrim | 4.3 | ±0.1 | High |
|  | *Phrynorhombus norvegicus* | Norwegian topknot | 4 | ±0.60 | High |
|  | *Zeugopterus punctatus* | Topknot | 4 | ±0.66 | High |
| Gobidae | *Gobisculus flavescens* | Two-spotted goby | 3.2 | ±0.34 | Low |
|  | *Gobius niger* | Black goby | 3.3 | ±0.2 | Low |
| Syngnathidae | *Entelurus aequoreus* | Snake pipefish | 3.5 | ±0.44 | Low |
|  | *Syngnathus rostellatus* | Nilsson's pipefish | 3.7 | ±0.40 | Mid |
|  | *Syngnathus acus* | Greater pipefish | 3.3 | ±0.2 | Low |
| Clupeidae | *Clupea harengus* | Atlantic herring | 3.4 | ±0.1 | Low |
|  | *Sprattus sprattus* | European sprat | 3 | ±0.07 | Low |
| Cottidae | *Myoxocephalus scorpius* | Shorthorn sculpin | 3.9 | ±0.0 | Mid |
|  | *Taurulus bubalis* | Longspined bullhead | 3.6 | ±0.52 | Mid |
| Liparidae | *Liparis montagui* | Montagu’s seasnail | 3.5 | ±0.57 | Low |
| Soleidae | *Solea solea* | Common sole | 3.2 | ±0.1 | Low |
| Gasterosteidae | *Spinachia spinachia* | Fifteen-spined stickleback | 3.5 | ±0.37 | Low |

**Table S2** (continued)

| Anguillidae | *Anguilla anguilla* | European eel | 3.6 | ±0.2 | Mid |
| --- | --- | --- | --- | --- | --- |
| Lotidae | *Ciliata mustela* | Fivebeard rockling | 3.5 | ±0.3 | Low |
| Cyclopteridae | *Cyclopterus lumpus* | Lumpsucker | 3.9 | ±0.0 | Mid |
| Pholidae | *Pholis gunnellus* | Rock gunnel | 3.5 | ±0.46 | Low |
| Belonidae | *Belone belone* | Garfish | 4.2 | ±0.4 | High |
| Zoarcidae | *Zoarces viviparus* | Eelpout | 3.5 | ±0.49 | Low |
| Agonidae | *Agonus cataphractus* | Hoocknose | 3.4 | ±0.3 | Low |
| Callionymidae | *Callionymus lyra* | Common dragonet | 3.3 | ±0.38 | Low |
| Trachinidae | *Trachinus draco* | Greater weever | 4.2 | ±0.71 | High |
| Salmonidae | *Salmo trutta* | Sea trout | 3.4 | ±0.1 | Low |

**Table S3** Summary of Shannon-, Simpson- and evenness indices for sample sites in both national parks for all years, divided into 11 or 12 clusters based on north-south gradient, where 1 is located in the northernmost part, and 12 is located in the southernmost part.

| **National park** | **Cluster** | **Shannon index (H')** | | | **Simpson's index** | | | **Evenness (J)** | | |
| --- | --- | --- | --- | --- | --- | --- | --- | --- | --- | --- |
|  |  | **2017** | **2018** | **2019** | **2017** | **2018** | **2019** | **2017** | **2018** | **2019** |
| **Færder national park** | 1 | 2.04 | 1.84 | 2.22 | 0.8 | 0.74 | 0.84 | 0.77 | 0.65 | 0.78 |
|  | 2 | 1.83 | 2.12 | 2.13 | 0.77 | 0.81 | 0.86 | 0.71 | 0.74 | 0.89 |
|  | 3 | 2.37 | 1.66 | 1.95 | 0.88 | 0.64 | 0.82 | 0.85 | 0.63 | 0.81 |
|  | 4 | 2.22 | 1.43 | 1.97 | 0.86 | 0.65 | 0.81 | 0.84 | 0.53 | 0.82 |
|  | 5 | 1.7 | 1.19 | 1.53 | 0.69 | 0.55 | 0.7 | 0.66 | 0.51 | 0.78 |
|  | 6 | 1.83 | 1.46 | 1.82 | 0.79 | 0.62 | 0.76 | 0.83 | 0.64 | 0.79 |
|  | 7 | 2.09 | 1.53 | 1.75 | 0.84 | 0.59 | 0.74 | 0.84 | 0.58 | 0.73 |
|  | 8 | 1.72 | 2.11 | 1.77 | 0.7 | 0.82 | 0.75 | 0.64 | 0.8 | 0.74 |
|  | 9 | 2.2 | 1.6 | 1.8 | 0.84 | 0.69 | 0.74 | 0.81 | 0.59 | 0.66 |
|  | 10 | 1.65 | 1.65 | 1.55 | 0.73 | 0.72 | 0.73 | 0.75 | 0.69 | 0.75 |
|  | 11 | 2.35 | 1.52 | 1.51 | 0.88 | 0.69 | 0.65 | 0.83 | 0.63 | 0.61 |
|  | 12 | 2.09 | 1.44 | 1.33 | 0.85 | 0.7 | 0.6 | 0.91 | 0.69 | 0.64 |
| **Ytre Hvaler national park** | 1 | 2.05 | 1.92 | 2.04 | 0.82 | 0.82 | 0.85 | 0.8 | 0.87 | 0.89 |
|  | 2 | 2.25 | 1.61 | 1.88 | 0.86 | 0.64 | 0.79 | 0.81 | 0.61 | 0.85 |
|  | 3 | 1.63 | 1.71 | 2.13 | 0.77 | 0.8 | 0.83 | 0.91 | 0.88 | 0.86 |
|  | 4 | 2.17 | 1.43 | 2.06 | 0.84 | 0.68 | 0.81 | 0.78 | 0.65 | 0.83 |
|  | 5 | 1.78 | 1.41 | 1.72 | 0.75 | 0.67 | 0.78 | 0.71 | 0.59 | 0.88 |
|  | 6 | 2.02 | 1.3 | 2.17 | 0.84 | 0.64 | 0.86 | 0.79 | 0.59 | 0.9 |
|  | 7 | 1.93 | 1.32 | 2.02 | 0.82 | 0.62 | 0.83 | 0.87 | 0.55 | 0.79 |
|  | 8 | 2.09 | 2.13 | 2.09 | 0.84 | 0.85 | 0.84 | 0.79 | 0.86 | 0.84 |
|  | 9 | 2.09 | 1.35 | 1.69 | 0.84 | 0.62 | 0.75 | 0.84 | 0.65 | 0.73 |
|  | 10 | 2 | 1.37 | 1.97 | 0.81 | 0.64 | 0.82 | 0.74 | 0.59 | 0.77 |
|  | 11 | 2 | 1.62 | 2.07 | 0.8 | 0.72 | 0.81 | 0.76 | 0.65 | 0.8 |
|  | 12 | 1.79 | - | 1.71 | 0.74 | - | 0.77 | 0.72 | - | 0.82 |

**Table S4** Summary of average Diversity indices, evenness and richness for east and west side of outer Oslo fjord and the Tvedestrand zones seascape for all sampling years.

| **Site** | **Year** | **Shannon index (H)** | **Simpson index (D)** | **Evenness** | **Richness** |
| --- | --- | --- | --- | --- | --- |
| **Oslo fjord west** | 2017 | 2.22 | 0.17 | 0.68 | 26 |
|  | 2018 | 2.02 | 0.22 | 0.61 | 27 |
|  | 2019 | 2.1 | 0.18 | 0.66 | 24 |
| **Oslo fjord east** | 2017 | 2.47 | 0.16 | 0.81 | 21 |
|  | 2018 | 1.89 | 0.25 | 0.63 | 20 |
|  | 2019 | 2.28 | 0.14 | 0.74 | 21 |
| **Tvedestrand** | 2017 | 2.22 | 0.16 | 0.68 | 26 |
|  | 2018 | 2.21 | 0.15 | 0.68 | 26 |
|  | 2019 | 1.82 | 0.24 | 0.56 | 26 |
|  |  |  |  |  |  |

**Table S5** Results from linear model predicting Shannon index using region and year as predictor variables (Shannon ~ Region * factor(Year)). Region FNP and year 2017 were set as reference levels (coded as zero) in the model.

| **Coefficients** | **Estimate** | **Std.Error** | **t-value** | **Pr(>\|t\|)** |  |
| --- | --- | --- | --- | --- | --- |
| (Intercept) | 1.14093 | 0.0281 | 40.597 | <2e-16 | *** |
| Region TVE | 0.06183 | 0.04325 | 1.429 | 0.1531 |  |
| Region YHNP | 0.03966 | 0.04056 | 0.978 | 0.3284 |  |
| factor(Year)2018 | -0.02408 | 0.04086 | -0.589 | 0.5558 |  |
| factor(Year)2019 | -0.09419 | 0.04102 | -2.296 | 0.0218 | * |
| RegionTVE:factor(Year)2018 | -0.00568 | 0.0579 | -0.098 | 0.9219 |  |
| RegionYHNP:factor(Year)2018 | -0.05938 | 0.06253 | -0.95 | 0.3425 |  |
| RegionTVE:factor(Year)2019 | -0.09851 | 0.05896 | -1.671 | 0.095 | . |
| RegionYHNP:factor(Year)2019 | -0.0772 | 0.05985 | -1.29 | 0.1974 |  |
| R^2^ | 0.03 |  |  |  |  |

**Table S6** Results from linear model predicting Simpson index using region and year as predictor variables (Simpson ~ Region * factor(Year)). Region FNP and year 2017 were set as reference levels (coded as zero) in the model.

| **Coefficients** | **Estimate** | **Std.error** | **t-value** | **Pr(>\|t\|)** |  |
| --- | --- | --- | --- | --- | --- |
| (Intercept) | 0.621134 | 0.01108 | 56.057 | <2e-16 | *** |
| RegionTVE | 0.022441 | 0.017052 | 1.316 | 0.1884 |  |
| RegionYHNP | 0.015649 | 0.015993 | 0.979 | 0.328 |  |
| factor(Year)2018 | -0.030493 | 0.016111 | -1.893 | 0.0586 | . |
| factor(Year)2019 | -0.038742 | 0.016173 | -2.395 | 0.0167 | * |
| RegionTVE:factor(Year)2018 | 0.012429 | 0.022826 | 0.544 | 0.5862 |  |
| RegionYHNP:factor(Year)2018 | -0.01925 | 0.024653 | -0.781 | 0.4351 |  |
| RegionTVE:factor(Year)2019 | -0.039041 | 0.023244 | -1.68 | 0.0933 | . |
| RegionYHNP:factor(Year)2019 | -0.009461 | 0.023598 | -0.401 | 0.6885 |  |
| R^2^ | 0.02 |  |  |  |  |

**Table S7** Results from linear model predicting Evenness using region and year as predictor variables (Evenness ~ Region * factor(Year)).

| **Coefficients** | **Estimate** | **Std.error** | **t-value** | **Pr(>\|t\|)** |  |
| --- | --- | --- | --- | --- | --- |
| (Intercept) | 0.899087 | 0.010107 | 88.954 | < 2e-16 | *** |
| RegionTVE | 0.001038 | 0.015555 | 0.067 | 0.94683 |  |
| RegionYHNP | -0.008851 | 0.014589 | -0.607 | 0.54416 |  |
| factor(Year)2018 | -0.087323 | 0.014697 | -5.942 | 3.64E-09 | *** |
| factor(Year)2019 | -0.034964 | 0.014753 | -2.37 | 0.01794 | * |
| RegionTVE:factor(Year)2018 | 0.039109 | 0.020821 | 1.878 | 0.06057 | . |
| RegionYHNP:factor(Year)2018 | 0.009685 | 0.022488 | 0.431 | 0.6668 |  |
| RegionTVE:factor(Year)2019 | -0.02595 | 0.021203 | -1.224 | 0.22122 |  |
| RegionYHNP:factor(Year)2019 | 0.061227 | 0.021526 | 2.844 | 0.00452 | ** |

**Table S8** Results from GLM (Cod presence predicted by region and fyke-nets used). Region Tvedestrand and year 2017 were set as reference levels (coded as zero) in the model. (Presence ~ Region * factor(Year)).

| **Coefficients:** | **Estimate** | **Std. Error** | **z value** | **Pr(>\|z\|)** |  |
| --- | --- | --- | --- | --- | --- |
| Intercept | -0.63 | 0.1769 | -3.562 | 0.000368 | *** |
| RegionFNP | 0.542 | 0.2309 | 2.348 | 0.01889 | * |
| RegionYHNP | 0.1892 | 0.2378 | 0.796 | 0.426223 |  |
| factor(Year)2018 | -1.7309 | 0.2932 | -5.902 | 3.58E-09 | *** |
| factor(Year)2019 | -2.2878 | 0.3451 | -6.629 | 3.38E-11 | *** |
| RegionFNP:factor(Year)2018 | 1.1163 | 0.3691 | 3.024 | 0.00249 | ** |
| RegionYHNP:factor(Year)2018 | 1.1201 | 0.3973 | 2.82 | 0.004809 | ** |
| RegionFNP:factor(Year)2019 | -1.4744 | 0.6939 | -2.125 | 0.033612 | * |
| RegionYHNP:factor(Year)2019 | 0.3106 | 0.5152 | 0.603 | 0.546529 |  |
| R^2^ | 0.16 |  |  |  |  |

**Table S9** Results from GLM (Cod >40 cm) presence predicted by region and fyke-nets used) for the three study areas. Region Tvedestrand and year 2017 were set as reference levels (Presence ~ Region * factor(Year)).

| **Coefficients:** | **Estimate** | **Std. Error** | **z value** | **Pr(>\|z\|)** |  |
| --- | --- | --- | --- | --- | --- |
| Intercept | -1.3083 | 0.1455 | -8.992 | < 2e-16 | *** |
| RegionFNP | -3.1915 | 0.5234 | -6.098 | 1.08 e -09 | *** |
| RegionYHNP | -3.0984 | 0.5237 | -5.917 | 3.28 e -09 | *** |
| factor(Year)2018 | -2.1619 | 0.3079 | -7.021 | 2.21 e -12 | *** |
| factor(Year)2019 | -2.3293 | 0.3266 | -7.131 | 9.98 e -13 | *** |
| RegionFNP:factor(Year)2018 | 3.4165 | 0.6589 | 5.185 | 2.16 e -07 | *** |
| RegionYHNP:factor(Year)2018 | 1.859 | 0.9232 | 2.014 | 0.0441 | * |
| RegionFNP:factor(Year)2019 | 1.8946 | 0.929 | 2.039 | 0.0414 | * |
| RegionYHNP:factor(Year)2019 | -11.8301 | 450.1 | -0.026 | 0.979 |  |
